# Alcohol Policy, Social Context, and Infant Health: The Impact of Minimum Legal Drinking Age

**DOI:** 10.3390/ijerph8093796

**Published:** 2011-09-23

**Authors:** Ning Zhang, Eric Caine

**Affiliations:** 1 Department of Community and Preventive Medicine, University of Rochester School of Medicine, 265 Crittenden Blvd., CU 420644, Rochester, NY 14642, USA; 2 Department of Psychiatry, University of Rochester School of Medicine, 300 Crittenden Blvd, Rochester, NY 14642, USA; E-Mail: eric_caine@urmc.rochester.edu

**Keywords:** alcohol policy, minimum legal drinking age laws, infant health

## Abstract

**Objective:**

The minimum legal drinking age (MLDA) was increased in the U.S. in the late 1980s in an effort to reduce intoxication-associated injuries, especially those related to motor vehicle accidents. This paper explores distal (secondary) effects of changing MLDA on indices of infant health, and whether changes in drinking behaviors or birth composition contributed to these effects.

**Methods:**

State- and year-fixed-effects models are used to analyze the relationship between MLDA, drinking behaviors, and birth outcomes. We studied the effects of different MLDA (age 18, 19, 20, or 21 years) when potential mothers were 14 years old by merging two population-based datasets, the Natality Detailed Files and the Behavioral Risk Factor Surveillance System between 1985 and 2002.

**Results:**

A MLDA of 18 years old (when potential mothers were 14 years old) increased the prevalence of low birth weight, low Apgar scores, and premature births. Effects were stronger among children born to black women compared with white women. Moreover, a younger MLDA was associated with an increasing proportion of very young and high school dropouts for black women. Furthermore, older MLDA laws at age 14 years decreased the prevalence of binge drinking among black women.

**Conclusions:**

Increasing the MLDA had longer term, distal impacts beyond the initially intended outcomes, specifically on birth outcomes (particularly among infants born to black women) as well as school drop-outs and binge drinking patterns among black young females. The older MLDA, intended initially to reduce problematic drinking behaviors, appeared to alter broader social contexts that influenced young women during their early childbearing years.

## 1. Introduction

Broadly applied changes in social or public health policies may yield unintended consequences, in addition to initially targeted changes [1]. A major example, the 18th Amendment to the U.S. Constitution (“Prohibition”) aimed to eliminate alcohol consumption, but served to spawn wide-spread illicit trade and to reinforce a criminal underground. In this vein, the changes in laws raising the minimum legal drinking age (MLDA) from age 18–20 years to 21 years to reduce motor vehicle accidents (MVAs) and related deaths, first at the state level and then nationally, established a series of “natural experiments” that offer the opportunity to examine the effects of different MLDAs on the health of infants who were born to young women that came into childbearing age when the MLDA varied between 18–21 years, and as well, intermediate social and behavioral outcomes that may have influenced infant health factors.

In the mid-1970s and early 1980s, organizations such as Mothers Against Drunk Driving argued to raise the MLDA after seeing increased MVA-related deaths associated with lowering the MLDA to 18 years old [2]. Congress enacted and President Reagan signed in 1984 the Uniform Drinking Age Act, with the MLDA set at age 21; this required withholding federal highway funds from states that failed to increase their MLDA [3]. By 1990, all states had complied. Literature has found that an older MLDA has both intended effects, *i.e.*, decreasing youth alcohol consumption and drunk driving, and unintended social and health benefits, such as decreasing high school dropouts, teen births, and the prevalence of sexually transmitted diseases [2].

Of particular relevance to our report, a recent publication noted that a MLDA of 18 years at the time of mothers’ conception year was associated with more adverse outcomes among births [4]. Drinking behaviors are repetitive and often habitual; thus we sought to examine the long-term impact of higher MLDAs. Particularly, we focused on a MLDA at the time when young women enter childbearing age, a proxy of alcohol availability at women’s younger teenage, and examine whether the MLDA at early teenage years would have improved the *average* birth outcomes to women who had children several years later. Following the study of Cook and Moore [5], we adopted 14 years as the “entry time” into the environment of alcohol control/availability associated with potential childbearing, as majority of teenage girls have entered puberty by then as well as having begun high school [6–8]. We sought to extend current research by examining whether MLDA was related to apparent behaviors or intermediate outcomes, which may be viewed as indicators of the broader social context in which potential mothers passed through their teenage years [5], and in turn may have been associated with indicators of infant health. Thus, we use in this study the MLDA as an indicator of potential alcohol-availability when the young women we studied were passing through their early childbearing years. We posited that an older MLDA was associated with a stricter alcohol-availability environment.

Studies already have found that alcohol consumption during pregnancy is associated with adverse consequences for the fetus [9]. Alcohol can pass through mothers’ bloodstream into the placenta, interfering with the fetus’ ability to get enough oxygen and nourishment for normal cell development in the brain and other body organs. [10] In addition, alcohol drinking is related to infant health. Drinking raises the likelihood of risky sexual behaviors, which accounts for a large proportion of unplanned pregnancies in the US [11–14]. These unplanned pregnancies augment the odds of poor-health infants through longer latency of pregnancy recognition and with delayed or lack of prenatal care [15].

Researchers have shown that social settings will influence individual behaviors. In an environment where alcohol control is lenient, adolescents tend to begin drinking relatively earlier [16,17]. People who begin drinking in early teenage years have a 40% higher risk of developing recurrent or habitual alcohol use or alcohol dependence some time in their lives than those who wait until age 21 years [18,19]. Moreover, pre-conception female drinking is a strong predictor of drinking and heavy drinking during pregnancy [20]. Hence, a stricter policy limiting access to alcohol may transfer benefits to the next generation by reducing drinking behaviors or their consequences among young women. Several supply-control policies could be candidates for assessing the alcohol-availability environment, such as alcohol taxes, the MLDA, or retail regulations. The MLDA has had sufficiently frequent changes associated with measurable variations, and published results studying the MLDA are consistent and robust in establishing the effectiveness of an older MLDA in reducing alcohol-related behaviors. Results from increasing alcohol taxes are mixed and other supply-control policies are not well-established [5]. Hence, we chose MLDA.

Overall, this study examined: (1) the relationship between alcohol-control environment during young women’s early childbearing years, indicated by the MLDA laws at their 14th birthday, and infant health indicators; (2) whether changes in birth composition, drinking behaviors, or formation of drinking habits contributed to change in infant health; and (3) whether racial differences relative to the above issues existed.

## 2. Method

### 2.1. Data

The study employs three data sources to understand the relationship between alcohol-controlled environment and infant health. The first one is the data extracted from the Vital Statistics Natality records from 1970 to 1992, census data that include all birth certificates, to estimate the relationship between MLDA laws at women’s age 14 and birth outcomes that occurred from ages 15–21. These data included infant information regarding birth weight, the 5-minute Apgar score and gestation length, and also mothers’ characteristics. The APGAR score is determined by evaluating the newborn baby on five criteria (Appearance, Pulse, Grimace, Activity, Respiration) on a scale from zero to two, then summing up the five values thus obtained. The resulting APGAR scores range from zero to 10. Low APGAR scores refer to scores below 7. We restricted the sample to young mothers who were 21 years old or younger at the time of delivery. In addition, since the MDA changed between 1970 and 1989, we further restricted the sample to women whose 14-years of age was between 1970 and 1989. Babies whose health indicators at birth were missing were dropped from the sample; if only one or two indicators were missing, the record was still kept in the sample. In the estimation, data were aggregated into cells according to residence, race/ethnicity, and year of mothers being aged 14 years. Panel A in Table 1 presents the summary statistics of Natality data. On average, 10.7% of infants were born with low birth weight (below 2500 g; LBW); 2% were extremely low (below 1500 g; ELBW); 3% of births had Apgar scores below seven, and 15% were premature births that were delivered before 37 gestational weeks. Infants born to black mothers had greater health burdens when compared to those of white mothers. About twice as many black infants were LBW (13.5% *vs.* 7.9%) or ELBW (2.7% *vs.* 1.5%). Black mothers had relatively more infants with low Apgar scores (3.5%) *versus* white mothers (2.4%). Among the black babies, 18% were born prematurely, compared to 11% of the white babies. The distribution of mothers’ education is very similar, but marital status differed greatly among white and black mothers: 35% of white mothers reported being married when their babies were born, while only 12% of black mothers did.

Despite of the Natality database’s rich information regarding infants’ health and their mothers’ characteristics, with its substantial underreporting, it is not a reliable source of data regarding drinking behavior. Table 1 also reported the prevalence of drinking during pregnancy from the Natality files. Less than 2% women had admitted that they drank during the pregnancy, which is much lower than other national numbers indicating that 10%–12% of American women drank during their pregnancy [21]. One possible reason is the data collection method, with a query about weekly use rather than monthly consumption [21]. For example, a woman who consumes alcohol at irregular intervals may not report herself as a drinker on a weekly basis. In addition, data were collected after the delivery in the hospital when an infant’s health status was already known; stigma regarding drinking during pregnancy may cause some women to lie about their alcohol consumption [21,22]. Finally, women with moderate to heavy use patterns may be fearful that accurate reporting may lead to questions about their parental fitness [22,23].

To circumvent these issues, we used data from another nationally representative survey to measure alcohol drinking among women, in keeping with the methods of other reports and researchers [24,25]. Results using the drinking information from the Natality data were not reported in this study and will be available upon request. Specifically we drew upon the Behavioral Risk Factor Surveillance System (BRFSS) from 1985 to 2002 to investigate women’s health behaviors. The BRFSS is a series of cross-sectional surveys, each of which is a representative sample of the non-institutionalized population of the United States. In each survey year, individuals were asked to report their drinking behaviors in a series of questions. Two variables were constructed accordingly: drinking prevalence and the incidence of at least five alcoholic beverages per occasion (*i.e.*, binge drinking) during the previous month. Similarly, the sample was limited to women who reached 14 years of age between 1970 and 1989 during which the MLDA varied across the states.

Panel B of Table 1 lists summary statistics of the BRFSS. Fifty-one percent (51%) of young women drank during the previous month of the survey, with an average of 2.7 alcoholic beverages per occasion. More white women (53%) reported drinking than black women (46%). Among white drinkers, 21% reported binge drinking at least once, whereas among black drinkers 25% of women had binge alcohol use. This finding was consistent with other national figures that more whites than blacks drink, but more black women are heavy or binge drinkers than their drinking white counterparts [19].

The data on MLDA laws come from the Distilled Spirits Council of the U.S. (DISCUS). We use the minimum drinking age at the estimated year of age 14 years as the indicator for the relevant policy regime. In some years, laws have split among different type of alcohol (beer, wine or liquor): beer was legal at age 18, but liquor was legal at age 21. In those instances, we coded the MLDA as age 18 years.

### 2.2. Specification

The following equation estimates the long-run effects of MLDA on infant health and drinking behaviors:

(1) $$Y_{st}=X_{st}\beta +M_{s,age-14}\rho +\mu_{s}+\lambda_{t}+{ɛ}_{st}$$

where *Y_st_* refers to outcomes (the incidents of low birth weight (LBW), low Apgar score, and premature birth of children, and the probability of drinking and binge drinking of mothers) when children were delivered at year t. *M_s,age–14_* refers to the state-specific variation of MLDA (18, 19, 20 or 21) when mothers were 14 years old. Since some states changed their MLDA within a year, this variable was constructed by identifying the MLDA policy that was in effect for the largest part of a calendar year when the child was delivered (for example, if one state raised the MLDA from age 19 to 20 in March of year when the women was 14 years old, the MLDA was set as age 20 for that year). The terms *μ_s_* and *λ_t_* capture the state- and year-fixed-effects. *ɛ_st_* is the mean-zero error term with finite variance. X includes mothers’ features including race, age, marital status and educational attainments. One would argue that even if the effects of the MLDA on birth outcomes or women’s behaviors are consistent with our hypothesis and significant, other factors such as macroeconomic conditions, not the MLDA, may drive the improvement. To remove this concern, the models include real *per capita* income representing state-specific macroeconomic environment during the birth year. Expected mothers’ smoking during pregnancy has been proved to be an important factor for poor infant health [26]. As the goal of this study aims to find out whether alcohol drinking during pregnancy has independent impacts on infant health, it is necessary to control for women’s smoking behaviors. Thus, an indicator of smoking during pregnancy was included in the model. Finally, some other alcohol policies have been found to influence on individuals’ behaviors as well as infant health [27]. To avoid omitted variable bias, we added real beer taxes (federal plus state) in this estimation since beer taxes changed between 1970 and 1989. In the estimation, the ordinary least square estimation was applied when the outcome variable was continuous, and a probit model was used when the outcome variable is dichotomous. For better interpretation, marginal effects were reported in probit model estimation.

## 3. Results and Discussion

### Primary results: The relationship between MLDA at age-14 and infant health

Table 2 presents the marginal effects of MLDA at women’s age-14 on birth outcomes. All models include state and year fixed effects. Among all mothers, a MLDA of age 18 when potential mothers were 14 years old increased the likelihood of having a child with LBW by 0.14 percentage points (LR *χ*^2^ = 1,160, df = 46, p < 0.001). This impact was largely associated with the difference between a MLDA of 18 and one of age 19 and age 21. The MLDA-19 decreased the LBW rate by 0.16 percentage points, while the MLDA-21 decreased the rate by 0.24 percentage points (LR *χ*^2^ = 11,605, df = 48, p < 0.001). Results were split by race in Panels B and C. The MLDA polices, however, predicted no change in LBW of children born to white mothers, while they were significantly predictive for children born to black women. For example, when black women faced a MLDA-18 at the age of 14, the probability of their children weighing less than 2,500 g at birth increased by 1.8 percentage points (LR *χ*^2^ = 7,605, df = 43, p < 0.001), and moving to an MLDA of 21 made the largest contribution to these effects by reducing the LBW 2.8 percentage points (LR *χ*^2^ = 7,611, df = 45, p < 0.001).

The relationship between the MLDA at women’s age of 14 and low Apgar scores (below 7) is shown in Columns 3 and 4. On average, living in a state with a more lenient drinking age policy (*i.e.*, MLDA-18) during their young teenage years increased the likelihood of mothers having an infant with low Apgar scores by 1.1 percentage points (LR *χ*^2^ = 15,605, df = 56, p < 0.001) when they became mothers at an older age when compared to other women. Exposure to any older age policy, *i.e.*, an MLDA-19, 20 and 21, decreased this probability by about 2 percentage points (LR *χ*^2^ = 15,108, df = 58, p < 0.001). Racial differences were still observed with this birth health indicator. Children born to white mothers who at the age of 14 lived in a state that adopted a MLDA-18 were 0.36% points (LR *χ*^2^ = 2,137, df = 46, p < 0.001) than those whose mothers lived in a state with older MLDA laws. Such effects were around 10 times larger for black women: children born to black mothers were 3.7% points (*χ*^2^ = 10,604, df = 48, p < 0.001) more likely to have low Apgar scores than their relative comparable groups. Age-21 laws were a significant driver to decrease the rate of low Apgar scores than other two age categories in either racial group. Along with the statistical significance, these results reveal a strong relationship between the drinking age laws at women’s young teenage years and probability of bearing a child with LBW or with low Apgar scores. Last, a MLDA of 18 predicted a 0.02 percentage points (LR *χ*^2^ = 10,304, df = 46, p = < 0.001) increase in the prevalence of premature births of women at their later life. These age policies, however, had no observed effects among white mothers but among black mothers. Children born to black mothers who at the age of 14 experienced an MLDA-18 were 0.6 percentage points (LR *χ*^2^ = 64,532, df = 43, p < 0.001) higher in being delivered pre-maturely than other children. States with a MLDA-21 predicted the largest decrease, roughly one percentage point (LR *χ*^2^ = 6,532, df = 45, p < 0.001) in premature birth for black mothers. In summary, results in Table 2 indicate that the drinking age policies had long-run impact on improving birth outcomes, particularly among those born to black women.

### Secondary Results: Pathways by which the MLDA may affect birth outcomes

How could the changes in MLDA laws affect birth outcomes? Tables 3 and 4 discuss two possibilities: the compositional shift or individual behavioral shift. The compositional shift refers that an older drinking age law may decrease the number of infants born to women with very young age or low education, as these women have higher risk of delivering a baby with poor health [26]. Table 3 shows that the compositional shifts occurred only among black mothers. On average, an exposure to MLDA-18 at age-14 increased the probability of very young mothers (*i.e.*, age 15–17) among blacks with 1.6 percentage point (LR *χ*^2^ = 64,695, df = 45, p < 0.001). Moreover, lenient alcohol availability, as reflected in the lower MDLA, increased the proportion of high school dropouts by 0.5 percent [F (72, 29927) = 1,286]. This effect was mainly associated with MLDA-21 laws, under which the proportion of mothers with less than a high school education dropped by 1.1 percentage point [F (75, 29914) = 1,365]. The age-specific legal access to alcohol had little impact on the composition of white mothers. While compositional shift is one possible explanation for the change in birth outcomes, results suggest that this shift only contributes to a very modest degree of improvement in birth outcomes for black mothers. No such effects are found among white mothers.

Using the individual-level data from BRFSS, Table 4 investigates whether variation of alcohol availability policies was related to behavioral changes that might have contributed to better birth outcomes. In particular, we examine the relationship between the probability of drinking and the context-defining MLDA laws when potential childbearing females reached age 14 years. Among the overall sample, the youngest MLDA regulations at women’s young teenage years increased the likelihood of drinking at their later age (*i.e.*, current survey-year age) with 0.15 percentage points (LR *χ*^2^ = 3,163, df = 68), and age-19 and age-21 partly explained these changes.

The stepwise change in MLDA laws had little effect on drinking among white women as the MLDA of age 21 years decreased the probability of alcohol drinking up to one percentage point, and had no significant effects on reducing binge drinking. In contrast, establishing the MLDA at age 21 years decreased the probability of alcohol drinking among black women by 2–8% points (LR *χ*^2^ = 3,169, df = 70). Moreover, MLDA differences during early teenage years predicted differences in binge drinking between young white and black women, with the younger MDLA associated with more binging among blacks. Those who lived in a state where the MLDA was age-18 were 2.2 percentage points (LR *χ*^2^ = 1,148, df = 63; p < 0.001) more likely to binge than those who lived in states with an older MLDA. Exposure to age-20 or -21 MLDA reduced the binge drinking by about 6.6 percentage points (LR *χ*^2^ = 12,052, df = 65).

These results indicate that the MLDA changed drinking behaviors among white and black women in a different way. For black women, a movement away from age-18 as the drinking age decreased overall drinking probability to a relatively modest degree and decreased binge drinking substantially more. Apparently, an increase in the legal drinking age modified the formation of habitual drinking by effectively reducing the availability of alcoholic beverages. For white women, an older MLDA modestly reduced overall drinking, but not binge drinking. This suggested that it was moderate white drinkers rather than heavy ones who were sensitive to changes in MLDA.

## 4. Conclusions

Our results showed that an older MLDA when girls turned age 14, which we postulated was related to the “alcohol environment” in which they spent most of their teen years, was related to improved infant health, particularly among those born to black women. These were associated with a lower incidence of LBW, low Apgar scores and premature births and hence, indicated that a stricter MLDA was related to improved infant health on average. This result is consistent with other studies since a stricter alcohol access policy is associated with lower risk for risky sexual behaviors, hence lower risk for unplanned births. [28,29]. In terms of public health, these estimated results suggested that, had there been a MLDA-21 when women in this study were teenagers, there would have been 128,800 fewer babies born with low birth weight, or 1,040,400 fewer babies born with low Apgar scores, or 18,400 fewer pre-mature babies (given that there were around 4 million infants born annually in the US). These results suggested that alcohol policies may have positive health consequences across generations through limiting formation of drinking behaviors.

Future work is needed to clarify how and why MLDA laws had different impacts among black and white young women. In addition, this study reflects a lack of data that simultaneously include reliable information regarding an infant’s health and his/her mother’s drinking behaviors. If such ideal data had existed, we could find whether the MLDA has a causal impact on birth outcomes through drinking behaviors, using two-stage least square models. This current study is unable to draw out this causality claim. Moreover, not all policies, such as expansion of Medicaid for pregnant women, were accounted for in this study. In addition, some other factors like insurance status, utilization and frequency of pre-natal care may influence infant health, but were not included in the analysis. Some of these variables are not available in the Natality data. Hence, the omitted variable bias may still exist. Despite these limitations, this study suggests that measures to improve the social context (*i.e.*, alcohol availability environment) can benefit the health of the next generation. It also offers an additional perspective to the recent debate whether the US should lower the age for alcohol consumption, prompted by current levels of under-age drinking among college-students [30,31]. Our data would suggest that lowering the MLDA would have adverse consequences well beyond those considered by the college presidents who promulgated this proposal.

## Figures and Tables

**Table 1:** Summary statistics.

	All		White		Black	
	
	Average Percentage	SD. Dev.	Average Percentage	SD. Dev.	Average Percentage	SD. Dev.
**A. Natality Data**						
white moms	51.05	49.01				
% born below 2500 g	10.68	7.54	7.96	2.534	13.54	9.70
% born below 1500 g	2.07	3.87	1.456	1.52	2.70	5.34
% Apgar score below 7	2.96	6.14	2.44	4.70	3.51	7.32
% pre-maturity birth	14.87	9	11.19	3.76	18.77	11.06
% mothers married	23.93	29.07	35.21	31.95	12.10	19.68
% mothers high school dropouts	59.30	31.55	60.66	30.58	57.85	21.48
% mothers high school grads	33.76	25.34	33.26	24.55	34.29	26.13
% mothers with some college	6.72	9.47	5.85	7.41	7.59	11.17
% mothers smoke during pregnancy	34.53	10.55	39.88	14.19	27.77	11.27
% mothers drank during pregnancy	1.77	4.54	1.92	5.44	1.4564	
N	11,051		7,448		4,603	
**B. BRFSS Data**						
% drink	51.11	50.00	52.65	50.00	45.72	50.00
# drink per time	2.71	2.31	2.73	2.33	2.67	2.21
% binge drink among drinkers	50.97	50.00	21.12	50.01	25.63	45.68
% high school dropouts	6.91	25.36	6.45	24.57	10.65	30.7
% high school graduate	31.21	46.33	30.39	46	37.8	48.49
% some college	30.15	45.89	30.06	45.84	30.91	46.21
% college or more	31.74	46.54	33.14	47.12	20.72	40.52
N	284,382		245,718		38,619	

**Table 2:** The effects of MLDA on birth outcomes.

	Low Birth Weight		Low Apgar Scores		Premature Birth	
**Columns**	1	2	3	4	5	6
**A. All Mothers**						
MLDA18	0.0014***		0.0112***		0.0002*	
p-value	<0.0001		<0.0001		0.0514	
MLDA19		−0.0016***		−0.0180***		−0.0002*
p-value		0.0020		<0.0001		0.0511
MLDA20		−0.0005		−0.0103***		0.0001
p-value		0.2174		<0.0001		0.2145
MLDA21		−0.0024***		−0.0182***		−0.0004***
p-value		<0.0001		<0.0001		0.0002
N	11,051	11,051	5,792	5,792	9,900	9,900
**B. White Mothers**						
MLDA18	0.0000		0.0036***		0.0000	
p-value	>0.1		<0.0001		>0.1	
MLDA19		0.0000		−0.0050***		0.0000
p-value		>0.1		0.0003		>0.1
MLDA20		0.0000		−0.0040***		0.0000
p-value		>0.1		0.0002		>0.1
MLDA21		0.0000		−0.0061***		0.0000
p-value		>0.1		<0.0001		>0.1
N	7,448	7,448	3,471	3,471	5,677	5,677
**C. Black Mothers**						
MLDA18	0.0181***		0.0372***		0.0061**	
p-value	<0.0001		<0.0001			
MLDA19		−0.0202		−0.0257***		−0.0056
p-value		0.0071		<0.0001		0.2102
MLDA20		−0.0073		−0.0368***		−0.0027
p-value		0.2671		<0.0001		0.5029
MLDA21		−0.0283***		−0.0498***		−0.0095***
p-value		<0.0001		<0.0001		0.0075
N	4,603	4,603	2,321	2,321	4,223	4,223

Notes: 1. Marginal effects are presented in the Table. 2. The dataset is the aggregated Natality files 1970–1992. Sample is restricted to mothers younger than 21 years old. Models include controls such as mothers’ education, age, marital status, smoking behaviors during pregnancy, real income per capita and real beer taxes (federal plus state level). 3. The numbers in the column headings represent the type of models we refer to. For instance, Column 1 refers to the model that includes MLDA-18 as well as other co-variates. All models include state and year fixed effects. Marginal effects are reported and standard errors in parentheses according to probit estimation. Sample is restricted to mothers younger than 21 years old. 4.

* Statistically significant with a p-value <0.10,

** Statistically significant with a p-value <0.05,

*** Statistically significant with a p-value <0.01.

**Table 3:** The effects of MLDA on compositional change of births.

	Mother Age 15–17		% High School Dropouts	
	1	2	3	4
**A. All Mothers**				
MLDA18	0.0069*		0.0004	
p-value	0.1682		0.8501	
MLDA19		−0.0028		0.0016
p-value		0.6404		0.5252
MLDA20		−0.0253		0.0005
p-value		0.0046		0.8892
MLDA21		−0.0053		−0.0028
p-value		0.9286		0.2477
N	11,051			
**B. White**				
MLDA18	−0.0015		−0.0049	
p-value	0.8191		0.0471	
MLDA19		0.0078		0.0048
p-value		0.3128		0.0937
MLDA20		−0.0102		0.0057
p-value		0.3519		0.1606
MLDA21		−0.0012		0.0048
p-value		0.8736		0.0937
N	7,448			
**C. Black**				
MLDA18	0.0160**		0.0056*	
p-value	0.0336		0.0958	
MLDA19		−0.0139		−0.0014
p-value		0.1161		0.6405
MLDA20		−0.0047		−0.0042
p-value		0.713		0.4629
MLDA21		−0.0093		−0.0106***
p-value		0.2856		0.0108
N	4,603		4,603	

*Notes*: 1. Marginal effects are presented in the Table. 2. The dataset is the aggregated Natality files 1970–1992. Sample is restricted to mothers younger than 21 years old. Models include controls such as mothers’ education, age, marital status, smoking behaviors during pregnancy, real income per capita and real beer taxes (federal plus state level). 3. The numbers in the column headings represent the type of models we refer to. For instance, Column 1 refers to the model that includes MLDA-18 as well as other co-variates. All models include state and year fixed effects. Marginal effects are reported and standard errors in parentheses according to probit estimation. Sample is restricted to mothers younger than 21 years old. 4.

* Statistically significant with a p-value <0.10,

** Statistically significant with a p-value <0.05,

*** Statistically significant with a p-value <0.01.

**Table 4:** The MLDA and the drinking behaviors.

	Drinking (0/1)		Binge Drinking (0/1)	
	1	2	3	4
**A. All Mothers**				
MLDA18	0.0015		−0.0095	
p-value	0.6048		0.0102	
MLDA19		−0.0057*		−0.0003
p-value		0.0880		0.9364
MLDA20		−0.0042		0.0404
p-value		0.4206		0.6541
MLDA21		−00056*		0.0149***
p-value		0.1543		0.0039
N	284,382		154,080	
**B. White**				
MLDA18	0		−0.007	
p-value	>0.1		0.3846	
MLDA19		−0.0046		−0.0021
p-value		0.1945		0.6486
MLDA20		−0.0057		0.056
p-value		0.2928		0.4037
MLDA21		−0.0085***		0.0257
p-value		0.0421		0.0974
N	245,718		140,328	
**C. Black**				
MLDA18	0.0015		0.0219***	
p-value	0.8421		0.0200	
MLDA19		−0.0282		−0.0046
p-value		0.0482		0.9603
MLDA20		−0.0823***		−0.0659***
p-value		0.0006		<0.0001
MLDA21		−0.0234**		−0.0656***
p-value		0.1256		<0.0001
N	38,619		13,752	

*Notes*: 1. Marginal effects are presented in the Table. 2. The dataset is the aggregated Natality files 1970–1992. Sample is restricted to mothers younger than 21 years old. Models include controls such as mothers’ education, age, marital status, smoking behaviors during pregnancy, real income per capita and real beer taxes (federal plus state level). 3. The numbers in the column headings represent the type of models we refer to. For instance, Column 1 refers to the model that includes MLDA-18 as well as other co-variates. All models include state and year fixed effects. Marginal effects are reported and standard errors in parentheses according to probit estimation. Sample is restricted to mothers younger than 21 years old. 4.

* Statistically significant with a p-value <0.10,

** Statistically significant with a p-value <0.05,

*** Statistically significant with a p-value <0.01.
